# Diversity of Orchid in *Abies nephrolepis* Nature Reserve in Shanxi, China

**DOI:** 10.1002/ece3.70433

**Published:** 2024-10-14

**Authors:** Xiaojiang Chen, Jiajia Li, Wenjiao Wei, Qianru Fu, Qingrong Zheng, Meihong Li, Xing Li

**Affiliations:** ^1^ Department of Biology Xinzhou Normal University Xinzhou China; ^2^ College of Biological Science and Engineering North Minzu University Yinchuan China; ^3^ Lanzhou University Lanzhou China; ^4^ Ocean University of China Qingdao China; ^5^ Department of Geography Xinzhou Normal University Xinzhou China; ^6^ Administration of Shanxi Abies Nephrolepis Provincial Nature Reserve Xinzhou China; ^7^ Inner Mongolia Engineering Research Center for Water‐Saving Agriculture Inner Mongolia Normal University Hohhot China

**Keywords:** *Abies nephrolepis* nature reserve, altitude, habitat, orchid, species diversity

## Abstract

*Abies nephrolepis* Nature Reserve has complex habitats and rich species diversity but lacks systematic ecological surveys. We focused on Orchid in *Abies nephrolepis* Nature Reserve, investigated and analyzed Orchid diversity and changes in community structure according to the characteristics of the alpine valleys in the study area, in terms of altitude gradient and habitat type, using the sample line method and the quadrat method. The results showed that 11 genera and 13 species of Orchidaceae were found in the survey, among which the species richness of *Malaxis monophyllos* was the highest, *Neottia puberula* was the second highest, and the species richness of *Tulotis ussuriensis* was the lowest, and the species with relative plurality ≥ 10% were *Malaxis monophyllos* (51.69%), *Neottia puberula* (14.77%), and *Cypripedium guttatum* (11.15%). The results of diversity analysis showed that Orchidaceae species were rich and the diversity index were the highest in the middle altitude area of 1950–2250 m; the distribution of Orchidaceae in the low altitude area was obviously clustered, and the similarity with the high altitude area was low. With the increase in altitude, the number of species showed an increase and then a decrease, showing a single‐peak state, which was in line with the theory of “Intermediate altitude expansion hypothesis”. The species and number of Orchidaceae in different habitats of *Abies nephrolepis* Nature Reserve also varied considerably, with Shannon‐Wiener diversity index being the highest in EV4 (*Abies nephrolepis* + *Picea meyeri* + *Picea wilsonii* + *Larix gmelinii* var. *principis‐rupprechtii*—*Lonicera hispida*—*Carex lanceolata* community) and EV5, and the smallest in EV1 habitats, Pielow evenness index being the highest in EV5 habitats and the lowest in EV1 habitats, and Simpson's index being the highest in EV5 habitats and the lowest in EV1 habitats. This study provides a scientific basis for strengthening regional monitoring of species diversity and protecting biodiversity.

## Graphical abstract

Nature reserves are important bases for global biodiversity research and conservation. To conduct in‐depth research on biodiversity conservation and to maintain the ecosystems on which human beings depend for their survival, we took Abies nephrolepis Reserve as the object of research, conducted a special survey on orchids, and analyzed the characterization of the diversity of orchids in different habitats and on different altitudinal gradients, and obtained very good results.

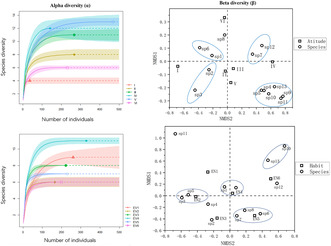

## 1 Introduction

Orchidaceae is a relatively special taxon in the process of plant systematic evolution, and its biodiversity and regional characteristics are one of the hotspots of ecological research (Jin, Xiang, and Jin 2015). Currently, there are ~25,000 orchid species recorded worldwide, with new species being recorded every year as research progresses (Kang et al. 2022). Orchidaceae is a special group in the process of plant systematic evolution, and many orchids have high ornamental and medicinal values, which are frequently exploited in different parts of the world and are subject to the limitations of germination, mycorrhizal specificity and pollinator specificity, resulting in the endangerment of many species, which has become the “flagship” group in the conservation of plants (Ye et al. 2022). Since the 20th century, the monitoring and study of orchid plant diversity has received much attention (Qin et al. 2012). The State Forestry and Grassland Administration of China launched a special survey on national wild orchid plant resources in 2018, established a scientific survey system that can be reviewed, expanded and monitored, and systematically and comprehensively grasped the ontogenetic status, diversity, geographic distribution of populations, degree of endangerment and current conservation status of wild orchid plant resources nationwide, as well as conducted a comprehensive and in‐depth study and assessment and analysis of the factors that have caused the endangerment of wild orchid plant resources. Currently, there are fewer studies investigating orchid diversity and analyzing its influencing factors from forest ecosystems to grassland ecosystems (Zhou et al. 2022; Zheng et al. 2023), e.g., habitat differences and changes in altitudinal gradients can significantly change orchid abundance (Kang et al. 2022), and habitat changes are the determinants of the temporal and spatial distribution pattern of orchids. Habitat variation is a determinant of the spatial and temporal distribution pattern of orchids, and more orchid species are distributed in habitats with high species richness than in habitats with low species richness (Tian and Xing 2008).

The Wutai Mountains are a branch of the northern Taihang Mountains, with a wide range of elevation gradients and a maximum elevation of 3061 m, making them the roof of North China (Ru and Zhang 2000). Its complex topographic and geomorphological features and diverse environmental conditions have nurtured rich vegetation and biodiversity (Jiang, Huang, and Liu 2009), and it is also one of the biodiversity hotspots in northern China. The Wutai Mountains are located in three ecological intertwined zones, including the transition zone between the North China Plain and the Loess Plateau, the transition zone between the eastern monsoon zone and the northwestern arid zone, and the transition zone between farming and nomadic pastoralism, with a complex flora, and the study of endemics and distributional features of the flora in this region plays an important role in revealing the history of the formation of continental terrestrial flora (Cheng, Niu, and Hu 2014). The *Abies nephrolepis* Nature Reserve is located in the central part of the Wutai Mountain Range, and according to the data of the previous background survey, 865 species of seed plants are known, of which 286 are endemic to China (Ru and Zhang 2000); however, research reports on the distribution of the great variety of plant diversity are very limited (Liu et al. 2020), In particular, there is a gap in the study of orchid diversity and its distribution pattern. At present, there are only studies on the characteristics of C,N,P stoichiometry of *Abies nephrolepis* leaves, branches and roots at different altitude gradients in the Wutai Mountain area (Guo, Hu, and Zheng 2023), the understorey plant diversity of *Abies nephrolepis* forests in the reserve (Chen, Nie, and Zheng 2022), the structure of the soil protist community and the distribution pattern of the diversity in the altitude of the grassland soils of the subalpine mountains in the Wutai Mountain area (Luo, Liu, and Zhang 2023). This study investigates the diversity of orchids according to the characteristics of alpine valleys, vertical natural landscapes and three‐dimensional climates in the Wutai Mountain region. Based on a full review of the protected area's background survey data, the thesis team carried out a special survey, assessment and monitoring of orchids in the protected area in 2021, and answered three questions: (1) the current status of orchid species diversity in this reserve; (2) the changing pattern of orchid community diversity with elevation gradient and habitat type in this area; and (3) the assessment of endangered and protected species of orchids in this area. This will provide a scientific basis for monitoring and protecting the orchid diversity in the *Abies nephrolepis* Nature Reserve.

## 2 Materials and Methods

### 2.1 Overview of the Study Area

**FIGURE 1:**
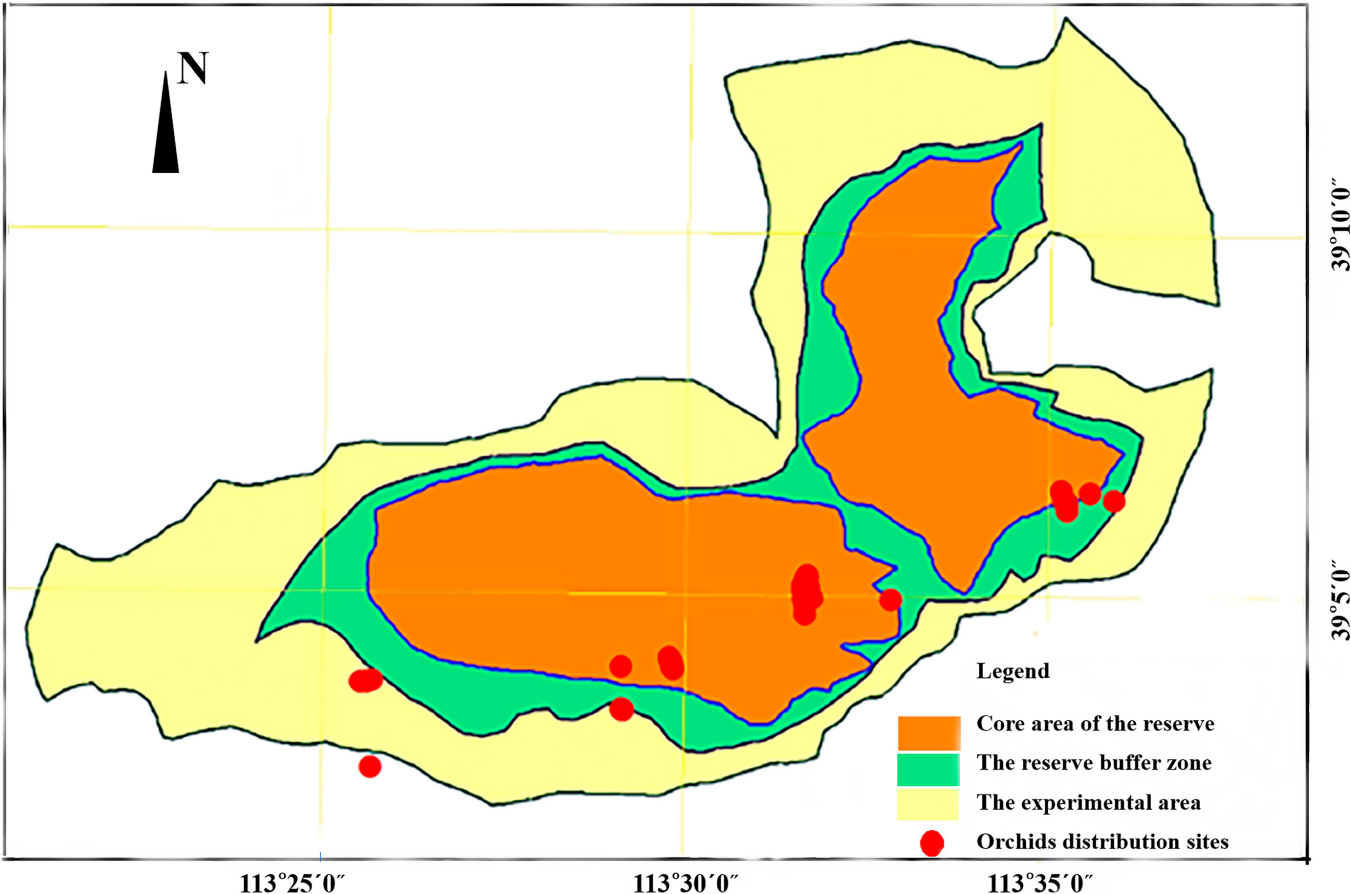
The main distribution range of Orchidaceae species in *Abies nephrolepis* Nature Reserve. Experimental zone: The outer edge of the buffer zone can be designated as an experimental zone, where activities such as scientific experiments, teaching practice, visits, tourism, domestication and breeding of rare and endangered wild animals and plants can be carried out.

Shanxi *Abies nephrolepis* Nature Reserve is located in the deep mountainous area of Wutai Mountain (E:113°20′55″–113°37′22″, N:39°02′04″–39°13′01″), and focuses on the protection of *Abies nephrolepis* species, endangered species of Orchidaceae such as *Epipogium aphyllum*, forest ecosystems and rare wild animals. The jurisdiction of the reserve contains the camping forest areas of Chantang, Niangnianghui, Erjialan, Dadonggou and Dagou, etc. (Figure 1). It is a comprehensive provincial nature reserve. The reserve is located in the temperate continental monsoon climate zone, with large vertical changes in temperature, and the temperature gradually decreases from low to high altitude. The average annual temperature is about 4°C, the extreme high temperature was 38°C, the extreme low temperature was −30°C, the frost‐free period is about 100d, the annual precipitation is concentrated in July to September, the annual precipitation is about 760 mm. Soil types include Wutai Mountain subalpine meadow soil, mountain forest brown loam, for‐land drenching brown soil, and mountain brown soil. *Abies nephrolepis* Reserve has high vegetation richness, the forest coverage rate reaches 30.65%, the regional climate is humid and warm climate zone, the main distribution range of the forest area is at the altitude of 1100 ~ 2600 m, the soil layer in the forest has high humus content, the soil pH is neutral and slightly acidic, and it is suitable for growth of Orchidaceae plants (Chen, Nie, and Zheng 2022).

### 2.2 Survey Methodology

From May to September 2021, within the jurisdiction of the reserve, five study areas were set up in the main distribution areas of orchids, namely, Chantang, Niangnianghui, Erjialan, Dadonggou, Dagou, and the field survey of orchids was carried out by using the sample line method and the sample method, and the survey was carried out according to the requirement of the special orchids survey to try its best to avoid interfering with the orchid and the neighboring habitats. The sample lines were selected according to the topography, vegetation and diversity of orchid in each study area, and the sample lines covered the main distribution area of orchid plants in the study area as much as possible, with a width of 20 m on both sides of the center line and a length of 150 m for each sample line, and 56 sample lines were designed in the five study areas; the sample squares were set up on flat or gently sloping land, and the spacing of the sample squares was ≥ 10 m. When setting up samples, there should be orchid plants in the samples, and try to include all orchid species in the study area, the number of species should cover at least 50% of the survey area, and for rare and endemic species, priority should be given to setting up samples or setting up samples individually, and the area of the samples should be 5 m × 5 m, and 156 samples were set up. We record the name of the sample line and sample plot location, elevation, vegetation community type, orchid species name and number of plants.

### 2.3 Data Analysis

Species identification was based on the Color Illustrated Catalog of Chinese Plants (Orchidaceae) (Chen 1999), the Flora of China (Vols., 17–19) (Lang and Chen 1999) and the Flora of Shanxi (Liu and Yue 2004). The degree of species endangerment was categorized into four grades of endangered (EN), vulnerable (VU), near‐threatened (NT), and concerned (LC) based on the Red List of China's Biodiversity (Volume of Higher Plants) (Ministry of Environmental Protection and Chinese Academy of Sciences 2013). The Alpha Diversity Index focuses on the number of species within a given habitat or localized homogeneous environment, also known as diversity within habitats. It measures the abundance and evenness of species within a given area. Shannon can be interpreted as the effective number of common species in a community; Simpson's diversity reflects the effective number of dominant species in a community, and Pielou's index is used to measure species evenness. Beta diversity refers to the dissimilarity of species composition between communities of different habitats along an environmental gradient or the rate of species turnover along an environmental gradient also known as between‐habitat diversity. The diversity index was calculated by the following formula (Hill 1970; Ma and Liu 1994; Whittaker 1972):

$$\text{Shannon}-\text{Wiener}\;\left(H\prime \right)\;\left(\text{Whittaker}\;1972\right):H^{\prime }=-\sum_{i=1}^{S}P_{i}\ln P_{i}$$

$$\text{Pielou}\;\left(E\right)\;\left(\text{Hill}\;1970\right):E=\frac{H΄}{\ln S}$$

$$\text{Simpson}\;\left(\lambda \right)\;\left(\mathrm{Ma}\;\text{and}\;\mathrm{Liu}\;1994\right):\lambda =\sum_{i=1}^{S}\frac{N_{i}\left(N_{i}-1\right)}{N\left(N-1\right)}$$

$$\text{Relative multiplicity}\;\left(P_{i}\right):P_{i}=\frac{N_{i}}{N}\times 100\%$$

where *S* is the number of species, *N* is the total number of individuals in the population, *N*_*i*_ is the total number of individuals in the population of the *i*th species, and *P*_*i*_ is the relative multiplicity.

The R software vegan package was used to analyze the species and number of Orchids in each study area. The samples were divided according to the 100 m altitude interval, and six different altitude segments were obtained (I: 1750–1850 m, II: 1850–1950 m, III: 1950–2050 m, IV: 2050–2150 m, V: 2150–2250 m, VI: 2250–2350 m.), and the number of Orchidaceae species in the different altitude segments was analyzed and multiplicity were analyzed. According to the classification of community types in the survey area, six different habitat types were obtained: EV1: *Larix gmelinii* var. *principis*‐*rupprechtii*—*Spiraea trilobata*—*Carex lanceolata* + *Fragaria orientalis* community, EV2: *Larix gmelinii* var. *principis‐rupprechtii* + *Picea wilsonii* + *Picea meyeri*—*Carex* spp. + *Conioselinum smithii* community, EV3: *Picea wilsonii* + *Picea meyeri*—*Spiraea trilobata*—*Carex* spp. community, EV4: *Abies nephrolepis* + *Picea meyeri* + *Picea wilsonii* + *Larix gmelinii* var. *principis‐rupprechtii*—*Lonicera hispida*—*Carex lanceolata* community, EV5: *Picea meyeri* + *Picea wilsonii* + *Betula albosinensis*—*Carex lanceolata* + *Fragaria orientalis* community, EV6: *Picea meyeri* + *Picea wilsonii* + *Betula platyphylla*—*Lonicera ferdinandi* + *Fragaria orientalis* community.

Using the R software iNEXT program package (Hsieh, Ma, and Chao 2016), which performs sparse extrapolation based on the relationship between the number of individuals and the number of species actually sampled, attention was paid to three metrics of qth‐order hill numbers: species richness (*q* = 0), Shannon (*q* = 1), and Simpson (*q* = 2). For each diversity metric, iNEXT used observed richness or incidence data to calculate diversity estimates for sparse and extrapolated samples and associated 95% (default) confidence intervals, estimate alpha diversity for orchid communities, and plot sparse and extrapolated (R/E) curves. Species richness is concerned only with the presence or absence of species, counting species equally regardless of their relative abundance, with larger values indicating greater species richness in the community; the Shannon‐Wiener index can be interpreted as the effective number of common species in the community. To assess the β‐diversity of Orchidaceae species composition among different factors (including six altitudinal gradients and six types of habitats), a non‐metric multidimensional scaling (NMDS) analysis based on Bray‐Curtis distances was carried out by using the R software vegan package based on the species richness at different factor levels, and the mutual distances of points were used to reflect the differences in the composition of the orchid community among different altitudes and levels of habitats. The number of species shared by orchids between elevations, habitats and seasons was quantified using the R software circlize program package, and the Jaccard community similarity index (Cs) was compared to see if there was a difference between the levels of the different factors, using the formula: Cs = *c*/(*a* + *b* − c) (Kitahara and Sei 2001), where, a is the number of species in community A, *b* is the number of species in community B, and *c* is the number of species common to both communities A and B. The Cs is defined as the number of species in the community A and B. Cs was defined as very dissimilar for 0–0.25, moderately dissimilar for 0.25–0.50, moderately similar for 0.50–0.75, and very similar for 0.70–1.00.

## 3 Results

### 3.1 Composition of Orchidaceae Plants Species

Through field surveys, 11 genera and 13 species of Orchidaceae plants were found in the *Abies nephrolepis* Reserve (Table 1), of which *Cypripedium guttatum* belongs to the subfamily Cypripedioideae, and the rest of the species belong to the subfamily Orchidoideae. There are 23 genera and 34 species of orchids recorded in Shanxi, and the survey found that the number of genera and species of orchids in *Abies nephrolepis* Reserve accounted for 52.17% and 40.63% of the genera and species of orchids in Shanxi Province respectively. There are 10 species of geophytic Orchidaceae plants in *Abies nephrolepis* Reserve, accounting for 76.92% of the total number of Orchidaceae plants species in the Reserve. There are three decaying types, accounting for 23.08% of the total number of Orchidaceae species in the reserve. The populations with higher numbers of plants were *Malaxis monophyllos* accounting for 51.69%, *Listera puberula* accounting for 14.77%, and *Cypripedium guttatum* accounting for 11.15%, the populations with lower numbers of plants were *Liparis japonica* accounting for 0.78%, *Coeloglossum viride* with 0.37%, *Herminium monorchis* with 0.37%, *Corallorhiza trifida* with 0.57%, *Tulotis ussuriensis* with 0.28%. The main components of the Orchidaceae plants in the reserve were *Malaxis monophyllos*, *Listera puberula* and *Cypripedium guttatum*. According to the IUCN (International Union for Conservation of Nature) rare and endangered classification, there were no extremely endangered Orchidaceae plants in the study area, and there were two kinds of endangered Orchidaceae species, namely, *Gymnadenia conopsea* and *Cypripedium guttatum*; there were three kinds of vulnerable orchid species, namely, *Malaxis monophyllos*, *Orchis roborovskii*, and *Liparis japonica*; and there were three kinds of near‐threatened Orchidaceae species, respectively, *Herminium monorchis*, *Corallorhiza trifida*, and *Tulotis ussuriensis*.

**TABLE 1:** Orchidaceae species in *Abies nephrolepis* Nature Reserve, Shanxi.

Species name	Genera name	The endangered levels	The relative abundance (%)	Life form
*Malaxis monophyllos*	*Malaxis*	VU	51.69	T
*Orchis roborovskii*	*Orchis*	VU	3.48	T
*Herminium monorchis*	*Herminium*	NT	2.27	T
*Platanthera chlorantha*	*Platanthera*	LC	6.46	T
*Coeloglossum viride*	*Dactylorhiza*	LC	1.35	T
*Gymnadenia conopsea*	*Gymnadenia*	EN	1.28	T
*Cypripedium guttatum*	*Cypripedium*	EN	11.15	T
*Listera puberula*	*Neottia*	LC	14.77	T
*Corallorhiza trifida*	*Corallorhiza*	NT	0.57	S
*Liparis japonica*	*Liparis*	VU	0.78	T
*Tulotis ussuriensis*	*Tulotis*	NT	0.28	T
*Neottia acuminata*	*Neottia*	LC	4.54	S
*Neottia camtschatea*	*Neottia*	LC	1.38	S

*Note:* The relative abundance, the proportion of the number of plants of a species in the study area to the total number of plants of all species in the area.

Abbreviations: EN, endangered; LC, concerned; NT, near threatened; T, Terrestrial; S, Saprophytic; VU, vulnerable.

### 3.2 Diversity Analysis of Orchids at Different Altitudes

**FIGURE 2:**
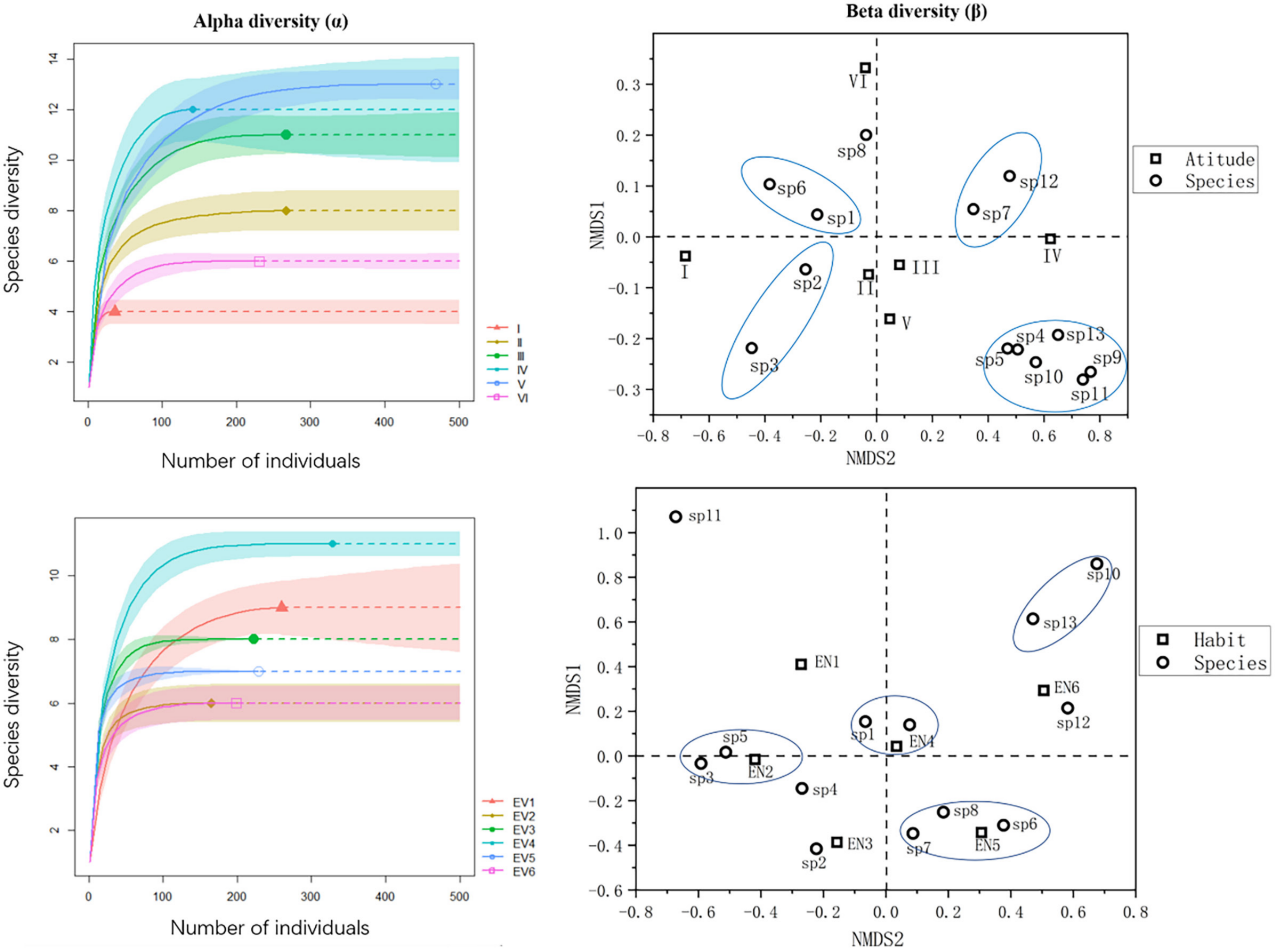
Alpha and beta diversity patterns of orchids among different altitudes, habitats in *Abies nephrolepis* Nature Reserve. Intervals around the line indicate the range of 95% confidence intervals.

The diversity index analysis is shown in Table 2, analyzing the species richness of orchids in different altitude gradients, and its richness value reached the maximum in the middle altitude region, i.e., the single‐peak change pattern, the maximum value of species richness was located in the sample site of the gradient of the altitude of 2050 to < 2150 m, and the species richness *S* = 13. There was a significant difference in the diversity of orchids in different altitude gradients, and it was also found in the sample site of the altitude of 2050 to < 2150 m. obvious differences. The highest species richness, Shannon index, Pielow evenness index and Simpson index of 12 and 13 Orchidaceae species were observed in the middle elevations of 1950 to < 2050 m and 2050 to < 2150 m (Table 2), indicating that the species richness and diversity of orchid species were the highest in this elevation range. The species richness and diversity of Orchidaceae were the highest in this altitude range. At the high altitude of 2150–2250 m, the number of Orchidaceae species and individuals was lower than that at the middle altitude, but the species richness, Shannon index and Simpson index were higher than those at the low altitude, whereas the species richness, Shannon index and Simpson index were the lowest at the low altitude of 1650 to < 1750 m (Table 2). The species richness, Shannon index and Simpson index were the lowest in the area of 1650 to < 1750 m at low altitude (Table 2). In terms of the spatial distribution pattern of β‐diversity, the species observed in the middle altitude area were obviously clustered, and spatially separated from the high elevation area with little overlap, and only Orchidaceae species such as *Malaxis monophyllos*, *Orchis roborovskii*, *Herminium monorchis*, and *Gymnadenia conopsea* were observed in the low elevation area; the species observed in the high altitude area were spatially dispersed, and the species observed in the high altitude area included *Malaxis monophyllos*, *Orchis roborovskii*, *Herminium monorchis*, *Gymnadenia conopsea*, *Cypripedium guttatum*, *Neottia puberula*, and *Neottia acuminata*. Orchidaceae species distributed in the middle altitude area were cross‐distributed with species distributed in the low altitude area, and some Orchidaceae species were overlapped with species distributed in the high altitude area, and rare Orchidaceae species such as *Corallorhiza trifida* and *Neottia camtschatea* can be observed (Figure 2; Table 2).

**TABLE 2:** Diversity index of orchids at different altitudes and habitats in *Abies nephrolepis* nature reserve.

Factors	*S*	*H′*	*E*	*λ*
Attitudes				
I	4	0.98	0.71	0.51
II	8	1.41	0.68	0.65
III	11	1.76	0.73	0.77
IV	12	2.00	0.80	0.83
V	13	1.42	0.55	0.57
VI	6	1.14	0.64	0.58
Habitats				
EV1	9	0.84	0.38	0.34
EV2	6	1.19	0.66	0.56
EV3	8	1.67	0.80	0.76
EV4	11	1.71	0.71	0.73
EV5	7	1.71	0.88	0.79
EV6	6	1.21	0.67	0.59

*Note: H*′: Shannon‐Wiener index, *E*: Pielou index, *λ*: Simpson index, *S*: Species richness.

### 3.3 Diversity Analysis of Orchids in Different Habitats

There were significant differences in the composition of orchids diversity among different community types in *Abies nephrolepis* Nature Reserve, with the highest species richness and Shannon index in EV4, highest Shannon index and lower species richness in EV5 habitat had the highest Shannon index, Simpson index and Pielow evenness index, and the species richness was smaller than that of EV4, EV1 and EV3; EV1 habitat had the lowest Simpson index and Pielow's evenness index; EV4 habitat had the highest number of orchids, but both Simpson index and Pielow evenness index were lower than EV5 habitat (Table 2). The spatial distribution pattern of β‐diversity indicated that the distribution of observed species was more concentrated in EV4 habitat and EV1 habitat, and the distribution of orchids species was more scattered in EV2 and EV6; the amount of overlap of orchids species between EV4 and the other habitats was small, while there was a larger portion of cross‐over and overlapping distribution of orchid species in the other community habitats (Figure 2). There were endemic orchids species in different habitats, e.g., *Liparis japonica* in EV6 and *Neottia camtschatea* in EV4.

### 3.4 Similarity Analysis of Orchids at Different Elevations and Habitats

In different altitudes and habitats, some well‐adapted and widely distributed orchid species had overlapping distributions; three Orchidaceae species were found in the six altitudinal gradients, namely, *Malaxis monophyllos*, *Orchis roborovskii*, and *Gymnadenia conopsea*. There were a total of four Orchidaceae species in the low and middle altitudes, a total of six Orchidaceae species in the middle and high altitudes, and only a total of three orchid species in the low and high altitudes. The maximum value of similarity coefficient of Orchidaceae species at different altitudes was between IV and V, with a similarity coefficient value of 0.92, which was extremely similar, and the minimum value was between I and V, with a similarity coefficient of 0.31, which was moderately dissimilar. The results showed that although some Orchidaceae species were shared at different altitudinal gradients, the similarity between the communities was low (Table 3).

**TABLE 3:** Community similarity index (Cs) for different altitudinal gradients and different habitats.

Factors	I	II	III	IV	V
Attitude (m)					
II	0.50				
III	0.36	0.73			
IV	0.33	0.54	0.77		
V	0.31	0.62	0.85	0.92	
VI	0.43	0.56	0.55	0.38	0.46

	Env1	Env2	Env3	Env4	Env5
Habitat					
Env2	0.36				
Env3	0.42	0.75			
Env4	0.54	0.55	0.73		
Env5	0.33	0.44	0.67	0.64	
Env6	0.36	0.20	0.27	0.42	0.44

Among the six habitats (EV1–EV6) in *Abies nephrolepis* Nature Reserve, only *Malaxis monophyllos* were common, of which E3 and E4 had the highest number of common species (eight species), E5 and E6 had four common species, and E2 and E3 had six common species. Although some Orchidaceae species were common to different habitats, the similarity of the communities was low. The composition of Orchidaceae communities at different altitudes and habitats had their own characteristics of adapting to the environmental conditions, and there were limited common species and low similarity of the communities (Table 3).

## 4 Discussion

A mapping survey on the status of wild orchid plant resources in *Abies nephrolepis* Nature Reserve area revealed that the unique geomorphology of crisscrossing peaks and valleys, three‐dimensional climate, and microhabitat differentiation has nurtured the rich species diversity in the area (Wang, Huang, and Wang 2022). Due to microtopographic redistribution of precipitation and soil nutrients, which constitute highly heterogeneous localized spatial environments, plants form a variety of community types under long‐term adaptation to different habitat conditions. Orchids are very sensitive to environmental and climate change and have been widely studied as indicator species (Xu et al. 2011). We examined habitat adaptations and characteristics of orchids in *Abies nephrolepis* Nature Reserve that varied with elevation gradients. Specifically, we explored the abundance and biodiversity of orchids at different altitudinal gradients and in different habitats. The results showed that the peak of orchid abundance in the reserve occurred in the mid‐elevation section, in line with the mid‐elevation expansion hypothesis. The results showed that compared with several neighboring nature reserves such as Luye Mountain Nature Reserve (11 wild orchid species were found), Pangquangu Nature Reserve (11 wild orchid species were found) and Heicha Mountain Nature Reserve (five wild orchid species were found) (the data of wild orchid species in each reserve were obtained from the reported information of the Forestry and Grasslands Bureau of Shanxi Province), *Abies nephrolepis* Nature Reserve had the most orchid species (13 wild orchid species were found). This indicates that the Nature Reserve is relatively rich in orchid resources in Shanxi Province.

Biodiversity distribution pattern is one of the hotspot issues in biodiversity research, in which the relationship between elevation gradient and biodiversity distribution is the main element of the hotspot issue (Hu et al. 2018). Because with the change in altitude gradient there will be a large difference in regional climate, there are many studies have shown that every 100 m of altitude gain, the temperature decreases by about 0.6°C, mountainous areas because of the increase in altitude, the temperature decreases, and there are often “four seasons in one mountain” regional climate characteristics (Jian et al. 2017). The results of many studies show that the distribution of seed plant species decreases with increasing altitude, and when reaching the middle altitude gradient, the species richness starts to decrease with increasing altitude, and the overall distribution shows a single‐peak distribution pattern. In other words, the distribution width of species on the altitudinal gradient has a “middle‐ground effect” (Tao, Zang, and Yu 2011). Altitude is considered to be an important factor influencing biodiversity (Liu et al. 2015). In mountain ecosystems, altitude is also an important factor influencing the composition of different habitats, with three patterns of species richness along the altitudinal gradient: increasing, decreasing or peaking at mid‐altitude (Wang, Huang, and Wang 2022). In this study, the highest orchid diversity was observed in *Abies nephrolepis* Nature Reserve at mid‐elevation, supporting the pattern of peak diversity at mid‐elevation. More studies have shown that the “in‐between effect” of species richness and vertical distribution patterns are more dependent on species with wider distribution widths. The mountain uplift hypothesis proposes that habitat fragmentation resulting from the process of mountain uplift promotes biodiversity differentiation (Xing and Ree 2017), and the effects of habitat and anthropogenic disturbances on orchid diversity have been widely noted, with protected areas with better habitats typically having higher species diversity, while habitats with more anthropogenic disturbances not only have fewer species but also have simpler community structures (Kang et al. 2022).

Mountainous areas are considered to be one of the higher biodiversity refuges due to the high environmental heterogeneity of mountainous areas (Lourenço et al. 2019). It has been shown that if the similarity coefficient of community structure at different altitudes is low, it indicates that the occurrence of orchids in this region has its own characteristics, which may be closely related to factors such as vegetation type, temperature, light and moisture. This suggests that vegetation type, precipitation and temperature are important ecological factors affecting the distribution of orchids, and these factors change with the altitudinal gradient (Zuo, Yang, and Li 2021). It has been found that with the change in altitude, a variety of environmental factors including hydrothermal conditions, light and humidity have changed considerably, forming different microclimatic zones within the mountainous region. There are studies of orchid abundance and density in the central Balkan Peninsula in altitudinal patterns, it is proposed that orchid abundance and density in the Balkan region is determined by mechanisms related to land area and habitat cover, in line with the species‐area relationship hypothesis (Djordjević et al. 2022). Some scholars have also pointed out that altitude also reflects the degree of anthropogenic disturbance of vegetation in a certain range (Li et al. 2008). The “single‐peak” pattern of orchid abundance in the study area as the altitude gradient increases is a wider distribution pattern, mainly because the low altitude area has more human disturbance, the high altitude area has lower temperature, and the middle altitude area has more suitable temperature, precipitation, and light suitable for most of the orchid growth. This is mainly due to the fact that the mid‐altitude region has more favorable temperature, precipitation and light suitable for the growth of most orchid species because of the strong disturbance of human activities in the low altitude region and the lower temperature in the high altitude region (Chang, Zhao, and Zhao 2004). The results of Djordjevi's study yielded a hump‐shaped distribution of orchid abundance and density at mid‐altitude. Our study similarly found that more species of orchids could be observed in habitats with different habitats at mid‐elevations with high diversity, while relatively fewer orchid species were surveyed at high elevations with low temperatures, low elevations with high levels of disturbance, and habitats with high levels of forest closure. This is because the middle altitude section is well developed and has the richest forest layer, thus possessing a microhabitat suitable for the growth of various orchids. It is worth noting that some studies have shown that the effect of altitude on orchid richness is not a single one, but rather a combined effect with topographic and geomorphological factors. Currently, there are various hypotheses to explain the single‐peak model of species diversity along the altitudinal gradient, such as the influence of climate (water and energy), area, geometric constraints, etc. In most mountainous regions, temperatures are high at lower altitudes, but the soil is of poor texture, with little humus layer and insufficient aeration because of rainfall. At higher altitudes, temperatures are low, humidity is high, light is abundant and aeration is good. The mid‐altitude section, on the other hand, has a complex and varied habitat, dominated by montane mixed coniferous and broad forests, and is typically characterized by moderate temperatures and humidity, good light and ventilation, and is suitable for the growth of most orchid species.

## 5 Conclusion

In this study, 11 genera and 13 species of orchids were found in *Abies nephrolepis* Nature Reserve. By analyzing the data from the field survey of orchids, the species richness and diversity indices of orchids in *Abies nephrolepis* Nature Reserve peaked in the mid‐altitude regions IV (1950 to < 2050 m) and V (2050 to < 2150 m), which is in line with the hypothesis of “intermediate altitude expansion”. Our study showed that the richness and diversity indices of orchid species under different habitat conditions were significantly differentiated, with the maximum values of species richness and biodiversity indices of orchids occurring in the EV4 and EV5. EV4 is *Abies nephrolepis* + *Picea meyeri* + *Picea wilsonii* + *Larix gmelinii* var. *principis rupprechtii—Lonicera hispida—Carex lanceolata* community, EV5 is *Picea meyeri* + *Picea wilsonii* + *Betula albosinensis—Carex lanceolata* + *Fragaria orientalis* community, and EV4 orchid species richness was higher than in habitat EV5. Our study also analyzed the similarity of orchids distributed in different altitudinal gradients; orchids distributed in different altitudinal gradients were less similar, with the highest degree of similarity of orchid species in the mid‐altitude regions IV and V. Orchid species distributed in different habitats were analyzed to be less similar. According to the results of the complex interrelationship between orchids diversity and habitat factors, altitude and habitat were the key factors affecting the distribution of orchids diversity, and protected areas need to pay attention to the high dependence of orchids on their native habitats when conserving orchids, and the implemented strategies should change with the change in altitudinal gradient, and need to set up different measures according to the adaptive characteristics of the orchid species and the habitat conditions. The strategies implemented should vary with the altitudinal gradient and should be tailored to the adaptive characteristics of orchid species and habitat conditions. In addition, our results suggest that changes in orchid habitat conditions may be reflected in patterns of orchid diversity distribution along elevational gradients. Future research areas should be pursued in terms of the effects of climate and other environmental factors on orchid species richness and diversity indices that vary along an elevational gradient in *Abies nephrolepis* Nature Reserve.

## Author Contributions

**Xiaojiang Chen:** conceptualization (equal), formal analysis (equal), investigation (equal), methodology (equal), resources (equal), software (equal), supervision (equal), writing – review and editing (equal). **Jiajia Li:** data curation (equal), investigation (equal), resources (equal), software (equal), writing – review and editing (equal). **Wenjiao Wei:** data curation (equal), investigation (equal). **Qianru Fu:** data curation (equal), investigation (equal). **Qingrong Zheng:** investigation (equal). **Meihong Li:** investigation (equal). **Xing Li:** conceptualization (equal), writing – review and editing (equal).

## Ethics Statement

This paper is not applicable for both human and/or animal studies.

## Conflicts of Interest

The authors declare no conflicts of interest.

## Data Availability

The first author and corresponding author agree with the editorial board to use the data only for the purpose of writing this article and not for reuse in other articles.
